# Clinical Outcome of a Novel Anti-CD6 Biologic Itolizumab in Patients of Psoriasis with Comorbid Conditions

**DOI:** 10.1155/2016/1316326

**Published:** 2016-11-02

**Authors:** Vinay Singh

**Affiliations:** Vibrance Skin Clinic, 412 Pearls Best Height II, Behind Max Hospital, Netaji Subhash Place, Pitampura, Delhi 110034, India

## Abstract

Psoriasis is a common, chronic, immune mediated, inflammatory disease of skin characterized by red patches enclosed with white scales and affects 2-3% of people in the world. Topical therapy, phototherapy, and systemic therapy were employed for management of disease from many last decades. However, long term uses of these agents are associated with unwanted effects and toxicities. Recently, Itolizumab has been developed as world's first anti-CD6 humanized monoclonal IgG1 antibody for the management of moderate-to-severe chronic plaque psoriasis in India. Here we are presenting the response indicated by Itolizumab in 7 Indian patients having moderate-to-severe psoriasis with severe comorbidities and who were intolerant/nonresponding to conventional therapies.

## 1. Introduction

Psoriasis is a common, chronic, immune mediated, inflammatory disease of skin characterized by red patches enclosed with white scales and affects 2-3% of people in the world [1]. Most common type of psoriasis is plaque psoriasis also known as psoriasis vulgaris which is found in 85–90% of psoriasis patients [2]. Disease severity is measured by body surface area involvement and classified into mild (≤2%), moderate (3–10%), and severe (>10%) [3]. Psoriasis affects social and psychological behaviors of patient due to its visibility and also has negative impact on patient's quality of life (QOL) [4]. Patients with moderate-to-severe psoriasis disease have increased association with comorbid conditions and may be linked to mutual pathogenic mechanism [5]. Psoriasis arthritis is a common psoriasis comorbidity, which affects about 20–30% of psoriasis patients. Psoriasis is related to comorbidities such as diabetes, obesity, depression, dyslipidemia, hypertension, Crohn's disease, and cardiovascular disease [6, 7]. The augmented risk of mortality is allied in psoriasis patients with the comorbid condition [6].

Topical therapy, phototherapy, and systemic therapy were employed for management of disease from many last decades. However, long term uses of these agents are associated with unwanted effects and toxicities [1]. The advances in molecular technology and improved understanding of psoriasis pathology have led to discovery of target specific biological therapies. These novel agents target specific cells and molecules involved in the development and maintenance of psoriatic plaques [8].

Recently, Itolizumab has been developed as world's first anti-CD6 humanized monoclonal IgG1 antibody for the management of moderate-to-severe chronic plaque psoriasis in India. It acts upstream by inhibiting the costimulation of T cells results in decreasing release of signature cytokines of Th1 and Th17 cells [9]. Here we are presenting the response indicated by Itolizumab in 7 Indian patients having moderate-to-severe psoriasis with severe comorbidities and who were intolerant/nonresponding to conventional therapies.

## 2. Case Presentation

Total of 7 patients (6 male and 1 female) with moderate-to-severe psoriasis and who were intolerant/nonresponding to conventional therapies were included in the present study. The details of patient such as demographic details, history of psoriasis, comorbid condition, and previous therapy are mentioned in Table 1. The included patients had comorbid condition of psoriatic arthritis, type 2 diabetes with hypertension, interstitial lung disease, alcoholic liver disease, and hypertension. The age range of patients was 35–63 years and weight range was 60–80 kg. All patients were screened for the clinical parameters like Psoriasis Area & Severity Index (PASI), Dermatology Life Quality Index (DLQI), and so forth to determine an ideal quantification for the biological therapy. Mantoux test was performed in every patient prior to receiving Itolizumab therapy to rule out latent tuberculosis. Liver function test (LFT), renal function test (RFT), Viral Markers for Hepatitis B and Hepatitis C and HIV, X ray Chest, complete blood count (CBC), and HbA1c were performed to find for any active infection. ECG was performed before starting of treatment in patient to evaluate any cardiac abnormality. All electrocardiograms of all the patients were found normal. Patients were found to be completely eligible for biologic therapy. Consequently, Itolizumab (1.6 mg/kg) was administered for each patient fortnightly for the first three months and monthly for the last three months as per the protocol of loading dose and maintenance of concentration in serum. PASI and DLQI score were recorded at baseline and on completion of therapy to evaluate the clinical improvement of the patients.

**Figure 1:**
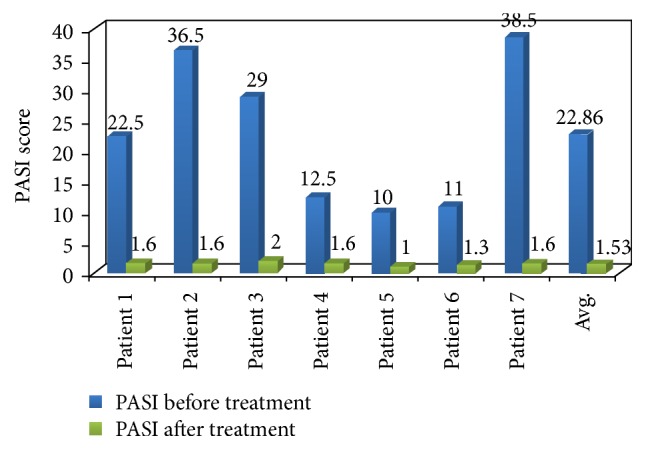
Effect of Itolizumab treatment on PASI score of patients.

**Figure 2:**
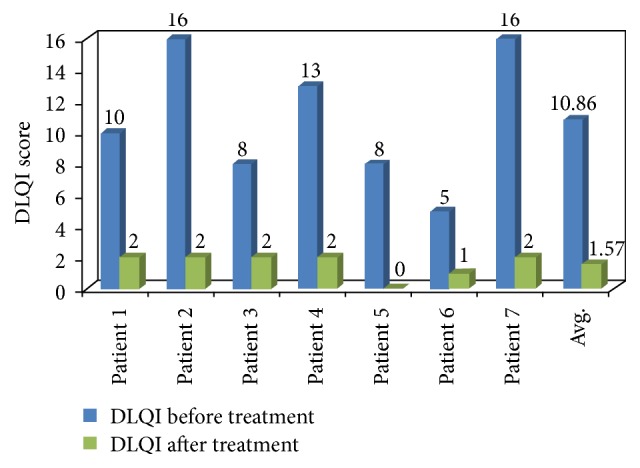
Effect of Itolizumab treatments on DLQI score of patients.

Patients included in the study were with an average age and weight of 49.8 years and 69.71 kg, respectively. Clinically significant improvement was seen in an entire group of patients exposed to Itolizumab therapy. The baseline mean PASI score was 22.8 which reduced to 1.53 after completion of Itolizumab treatment. Similarly mean baseline DLQI score also reduced from 10.8 to 1.57. Clinical improvement in the patient after Itolizumab treatment is given in Figures 1 and 2.

**Figure 3:**
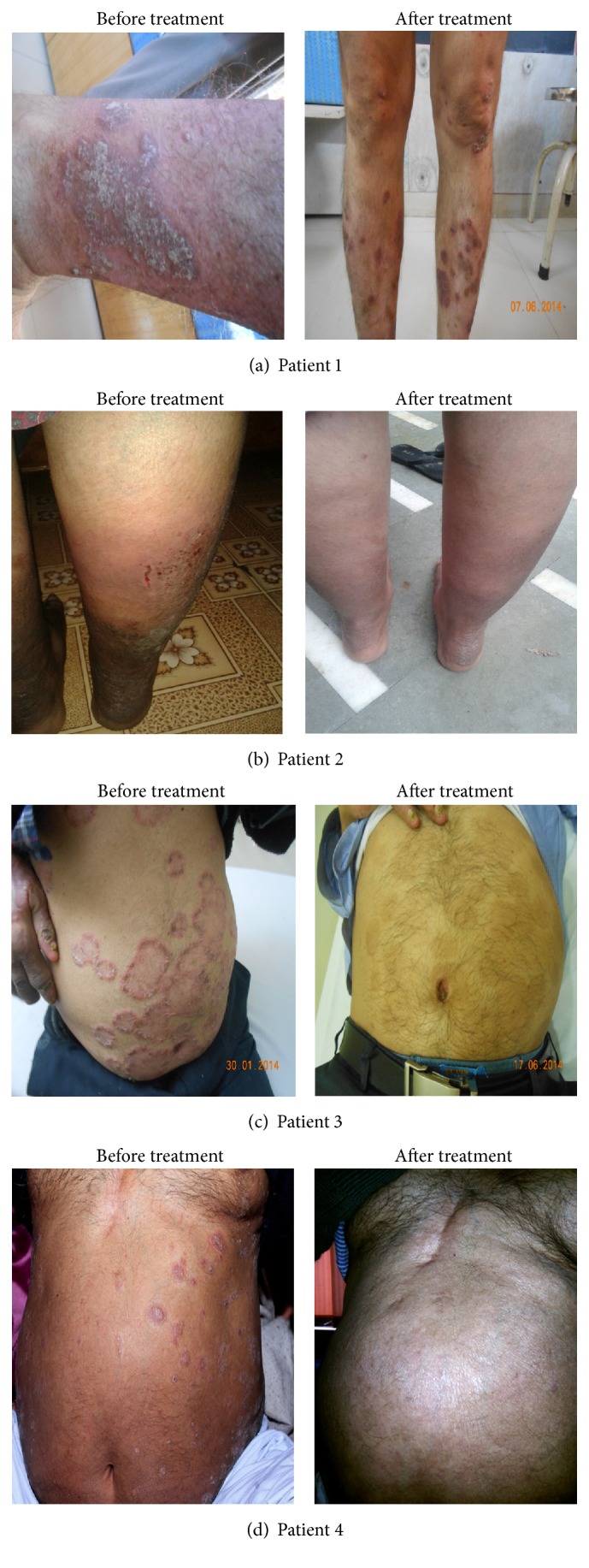
Effect of Itolizumab on psoriasis patients after treatment.

There was no significant weight gain observed in patients who received Itolizumab therapy. No signs and symptoms of cardiac abnormality were detected during and after treatment. There was overall improvement in psoriasis condition observed in all of the patients. Patient with psoriatic arthritis found great improvement in mobility of affected joints. Patients with other comorbid conditions did not observe any significant improvement in comorbid conditions. The effect of Itolizumab treatment on psoriasis patients is depicted in Figure 3.

## 3. Discussion

Psoriasis is emerging as a disease involving multiple systems and has a remarkable effect on patient's lives (physical, social, and psychological). The manifestation of comorbidity is vital in psoriasis patient since it is linked with significantly reduced duration and quality of life [10]. Therefore, there is an urgent need of effective treatment option for psoriasis. An introduction of biologic therapy has changed scenario of psoriasis treatment due to its highly target specific nature. Infliximab, Etanercept, and Adalimumab were employed for treatment of moderate-to-severe psoriasis [8]. In India, Itolizumab was approved for treatment of moderate-to-severe psoriasis which is also cost-effective therapy. Itolizumab acts by specifically binding to CD6 epitope, a surface protein of T cells, which is essential for optimum T-cell activation by the antigen-presenting cells. Formation of helper Th1 and Th17 cells and releasing of cytokines is an important phase in T-cell proliferation [9].

In this study, all patients with comorbid conditions treated with Itolizumab (1.6 mg/kg) exhibited significant improvement in clinical condition. Total of 5 patients attained PASI 90 response while 2 patients successfully achieved PASI 75 response after Itolizumab therapy. In a double blind, placebo controlled, phase III trial, Krupashankar et al. reported PASI 75 response achieved in plaque psoriasis patients after treatment of Itolizumab at week 12 as compared with placebo arm [11]. The mean baseline DLQI score was 10.8 reduced to 1.57 in the present study. Hence, the quality of life was also improved in all the patients after Itolizumab therapy.

The above assessment has proven that every individual of psoriasis showed a significant reduction in their PASI score and DLQI score. Comorbid conditions were not affected by Itolizumab injections. None of the patients experienced any severe kind of infusion reaction and/or infection during the treatment. No reactivation of latent tuberculosis was observed during and after the treatment. Itolizumab was found safe and well tolerated throughout the study.

## 4. Conclusion

In India, Itolizumab was found to be a safe drug of choice in an effective management of moderate-to-severe chronic plaque psoriasis with comorbid conditions. The efficacy proven on completion of therapy had also played a crucial role in improving QOL of all of those patients.

## Figures and Tables

**Table 1:** Demographic and comorbidity details of patients.

Patients	Height (cm)	Age (yr)	Body weight (kg)	History of psoriasis (yr)	Comorbid condition	Previous therapy
Patient 1	165.1	45	65	10	Psoriatic arthritis	Emollient, cyclosporine, and methotrexate
Patient 2	162.6	62	75	11	Type 2 diabetes with hypertension	Emollient and methotrexate
Patient 3	135.1	50	76	12	Interstitial lung disease	Emollient, cyclosporine, and methotrexate
Patient 4	160.0	63	80	10	Alcoholic liver disease	Emollient, cyclosporine, and methotrexate
Patient 5	135.1	35	60	6	Hypertension	Emollient and methotrexate
Patient 6	170.2	35	65	8	Hypertension	Emollient and methotrexate
Patient 7	152.4	59	67	13	Type 2 diabetes with hypertension	Emollient and methotrexate
